# Landslide type inference based on statistical analysis of a high-resolution digital elevation model in Gorce National Park, Poland

**DOI:** 10.1038/s41598-024-65026-z

**Published:** 2024-06-19

**Authors:** Robert Szczepanek, Mateusz Szczęch, Maciej Kania

**Affiliations:** Institute of Geological Sciences, Faculty of Geography and Geology, Jagiellonian University, 30-387 Krakow, Poland

**Keywords:** Natural hazards, Solid Earth sciences, Geomorphology

## Abstract

High-resolution digital elevation models are commonly utilized for detecting and classifying landslides. In this study, we aim to refine landslide detection and classification by analyzing the geometry of landslides using slope and aspect, coupled with descriptive statistics up to the fourth central moment (kurtosis). Employing the Monte Carlo method for creating terrain topography probability distributions and ANOVA tests for statistical validation, we analyzed 364 landslides in Gorce National Park, Poland, revealing significant kurtosis differences across landslide types and lithologies. This methodology offers a novel approach to landslide classification based on surface geometry, with implications for enhancing scientific research and improving landslide risk management strategies.

## Introduction

Landslide inventories are important to document the extent of landslide phenomenon in a region, in order to investigate the distribution, types, pattern, recurrence and statistics of slope failures^1^. When using geological data (lithological, structural, and bedding attitude), researchers use the geological units/formations shown in lithological or geological maps^2^. These are often identified using their regional or local names and characteristics, making it difficult to compare in different and distant areas^3^. For this reason, it seems important to describe the characteristics of landslide areas as universally as possible. One potential method is to utilize descriptive statistics. In the presented work, we seek to answer the question of whether it is possible to determine the geological conditions of landslide areas based only on the statistical analysis of the landslide's geometry.

### Landslides in the Outer Carpathians

Landslides are one of the elements of geomorphological relief, whose origin is related to the geological structure, and with neotectonic movements. These forms are a common element of the surface morphology of the Outer Carpathians, which models the relief of the slopes^4^. The landslides in the area presented in this research are earth-slide landslides, which are the most frequent landslides in Europe^5^ and worldwide^3^. The flysch formations, comprising the Outer Carpathians, and a highly dense network of faults and joints, facilitate landslides in this region. Similar conditions and triggers are also found in other flysch areas around the world^6,7^. Most extensive landslides are associated with wet interglacial and Holocene periods. In the Polish Carpathians, landslides are mainly triggered by antecedent precipitation and low-magnitude earthquakes^8,9^, and 93% of landslides have some form of contact with a river system^10^. In relation to the geological structure, landslides are often developed due faults, joints and mechanical contrast of sandstons and shales^11^. Typical for the Flysch Carpathians is the rotation of the slide packet of the rocks during movement.

### High-resolution terrain models from LiDAR data

Many methods are currently used to detect and monitor landslides. Light detection and range (LiDAR) is a method of laser scanning of a surface employed for the identification of landslides and the analysis of their morphological features. There are two main methods of laser scanning: Terrestrial Laser Scanning (TLS) and Airborne Laser Scanning (ALS). The TLS is powerful but time consuming and requires separate in-situ measurements of each landslide. On the other hand, ALS allows landslides to be monitored over larger areas at lower unit costs and was used for landslide mapping within the Polish National Landslide Counteracting System (SOPO), carried out by the Polish Geological Institute. High-resolution and multi-time Digital Elevation Models (DEMs) derived from LiDAR data are increasingly utilized to identify landslides^1,12–14^ and properly interpret landforms^15^. When manually determining the landslides from DEMs, it should be borne in mind that this is a process that is not devoid of subjectivity^16^.

### Geometric parameters of landslides

Among the topography parameters that best characterize landslide areas, the most frequently mentioned in the literature are those related to the slope, aspect, curvature and the topographic wetness index^17^. The slope angle is probably the main factor of stability as it affects the magnitude of both normal and shear stresses on the potential surface of failure^18^. With a slightly changing mean slope or aspect because of a landslide, their distributions may change significantly^19^. Hussin et al.^20^ presented a detailed analysis on sampling strategies for landslide raster models, whereby the shallow landslides in the Romanian Carpathians gave better success rates when sampled using the 50-m grid point method, while the scarp polygon method was better in predicting shallow landslides. The smallest uncertainty in the spatial representation of landslides is provided by the description of the area in the form of polygons, and thus characterized by a high level of detail^13^. The susceptibility model assessing the debris flow scarps in the Italian Alps had better success and prediction rates when using the entire scarp polygon compared to the other strategies^20^. Therefore, the polygonal method was chosen in this research as the basis for describing landslide geometry. Bernard et al.^21^ applied an automatic inventory of landslide sources and deposits based on 3D airborne LiDAR data for a 5 km^2^ area in New Zealand. However, to conduct reliable analysis for larger areas with various local conditions, their impact must be well described, preferably using statistical methods. Most of the studied landslide areas from Europe described in the literature^3^ have areas in the order of 10–1,000 km^2^.

### Statistical approach to landslide analysis

One of the first comprehensive studies of the quantitative statistical assessment of landslide geometry was the work of Carrara et al.^22^. They stated that landslide incidence, morphometry and typology are dependent on the interplay of land characteristics such as lithology and tectonic history. The analysis of variance method (ANOVA) was used in their study for statistical analysis. Keefer^23^ employed ANOVA to investigate how the occurrence of landslides correlates with slope steepness and lithology based on the 1989 Loma Prieta, California, event. This analysis was based on raster maps with a spatial resolution of 30 m. In the same spatial scale, a novel statistical analysis of landslides on a global scale was proposed by Lombardo et al.^24^, who developed the first statistical-based model in the literature able to provide information about the extent of the failed surface across a given landscape. Saito et al.^25^ utilized descriptive statistics (from the mean by standard deviation to the skewness and kurtosis) of elevation and slope angle for the construction of a decision-tree model of landslide. As a result, the mean slope angle, mode of slope angle and geology proved to be important variables for landslide susceptibility analysis. Yesilnacar and Topal^26^ employed the same descriptive statistics for landslide susceptibility mapping with a zone of 100 m in the upper part of the landslide (around the crown and flanks), regarded as an undisturbed, primary area. Work conducted in Turkey by Can et al.^27^ using interpretable machine learning models (XGBoost) showed that the most important factors in predicting landslide susceptibility are: lithology, altitude, the topographic wetness index, aspect and slope. Similar studies^28^ showed that the most important factors are the slope, surface area ratio and cross-sectional curvature. Based on periodically active landslides from the Polish Flysch Carpathians, Pawluszek^29^ found that the aspect and slope are the two first components in principal component analysis. To compile a reliable susceptibility map of Gdynia (Poland), only three independent controlling factors (slope angle, slope aspect and lithology) were sufficient from the 13 considered^30^. These reasons led us to limit the number of parameters to the most important ones (slope, aspect and lithology), at the expense of a broader in-depth analysis.

### Objective of the study

With the rapid adaptation of machine learning models for landslide vulnerability estimation^31^, little attention has been paid to the deeper statistical analysis of source materials in the form of high-resolution terrain models. The presented work is an attempt to analyze the geometric characteristics of landslides using statistical measures. The first objective of the present study was to develop a comprehensive inventory of landslides in the Gorce National Park, Poland. National parks in Poland are excluded from landslide monitoring; therefore, despite a few exceptions^4,32,33^, these areas are poorly explored. In landslide identification we focused on the contrast of morphology between the slope affected by landslide and an unaffected one. We formulated hypothesis of the research: it is possible to infer type of landslide solely based on statistical differences between morphology of landslide outline and its inside from high-resolution terrain models? To answer this question, we conducted a statistical analysis of changes in the landslide geometry depending on three types of lithology and four types of subsidence of geological strata. We also developed and applied an original, to the best of our knowledge, method of estimating the geometry of the terrain prior to a landslide by using the post-landslide outline and stochastic Monte Carlo method. The proposed method provides a solid statistical foundation for automated processing of topographic information on landslides in the era of big data.

### Study area

**Figure 1:**
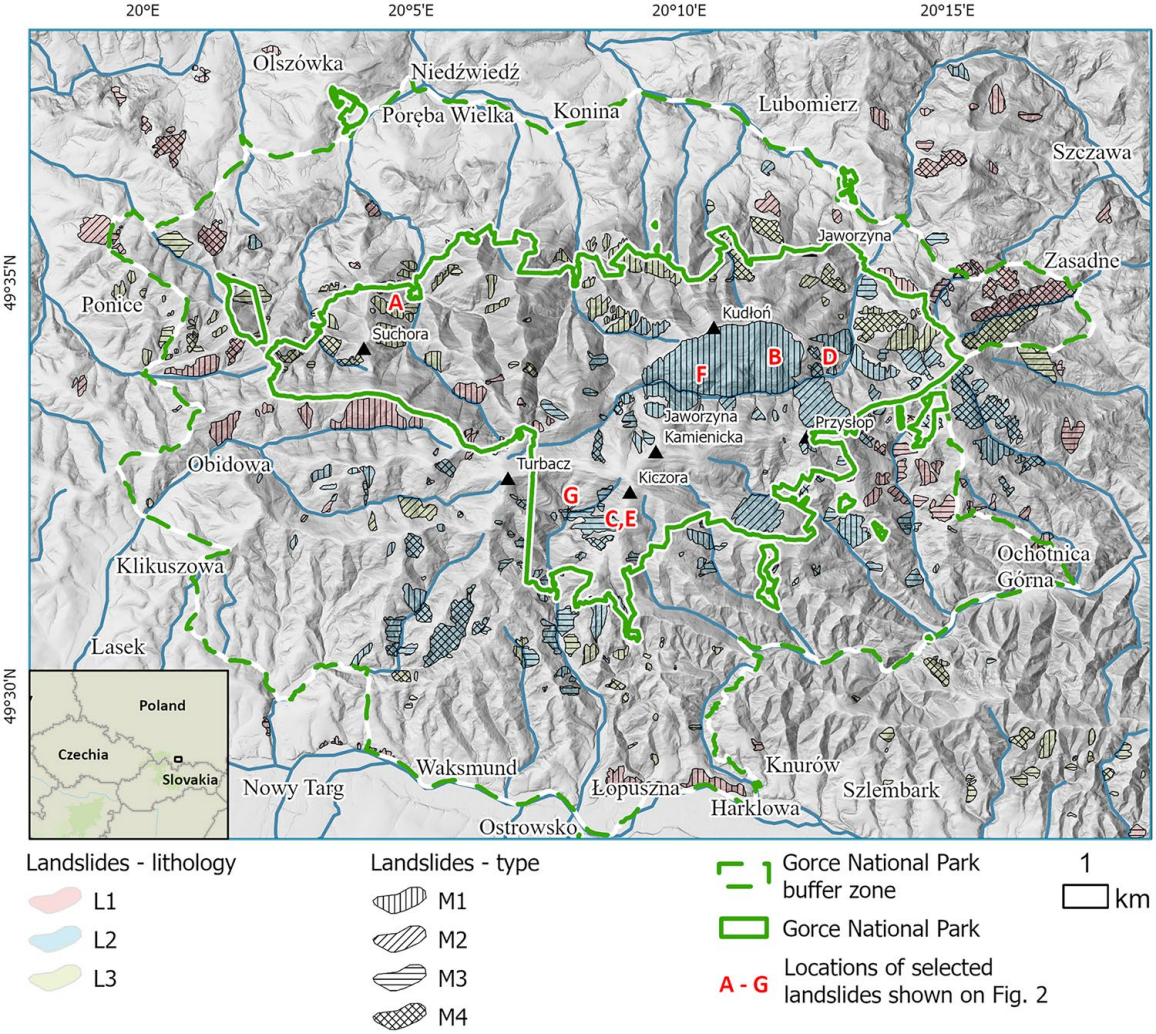
Spatial distribution of landslides identified in the Gorce National Park, Poland.

The Gorce Mts. are part of the Outer Carpathians and have the characteristic pattern of the ridges, which radiantly diverges from the highest peak (Turbacz 1310 m a.s.l.). The Gorce National Park with its buffer zone covers about 236 km^2^ (Fig. 1). The highest denivelations exceed 400 m. The southern edge of the study area has the characteristic of a foothills relief with denivelations up to 100 m. The slopes have convex or straight shape. One of the most important processes affecting the transformation of slopes, like elsewhere in the Outer Carpathians is the landslide. Over a dozen landslide depressions in the Gorce Mts. were investigated by Buczek^32^ and are dated to the cold and humid periods of the Holocene.

From a geological point of view, the study area is in the Outer Carpathians (Magura Nappe). The flysh sediments were formed as deep-sea siliciclastic deposits then folded and thrusted forming the nappes^34^. The flysch sedimentary series are categorized in formal lithostratigraphic units as shown in Table 1.^35,36^ . The general faulting direction is N-S ± ca. 20°. The faults are oftenform lineaments detectable on the DEM^35,37^. The general intensity of the lineaments increases from west to east, in consequence of approaching a local shear zone^37^.

**Table 1:** Landslide lithology categories of the Magura Nappe.

Category	Lithology type	Lithostratigraphic units	Number of identified landslides
L1	Thin- and medium-bedded sandstones and shales	Jasień Formation (Fm.), Malinowa Fm., Białe Fm., Ropianka Fm., Łabowa Fm., Beloveža Fm. and Malcov Fm	94
L2	Thick-bedded sandstones with intercalations of thin- and medium-bedded sandstones and shales	Szczawina Fm., Piwniczna Member and Kowaniec Member	139
L3	Thick-bedded sandstones	Poprad Member	131

**Figure 2:**
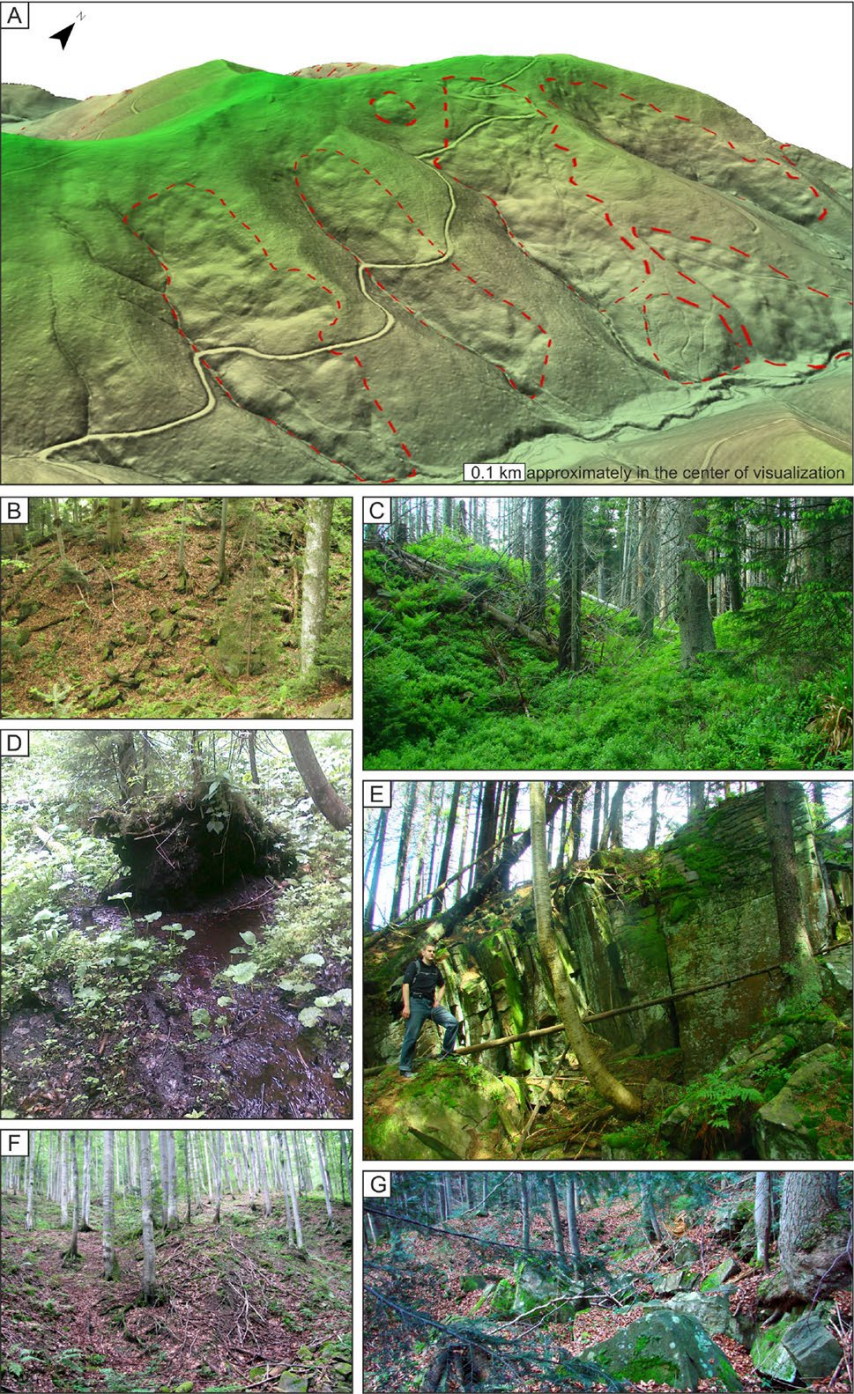
Selected landslides identified in the Gorce National Park, Poland. (**A**) visualization of DEM with landslides (red dotted outlines) – western slope of the Koninki Valley; (**B**) colluvium of the landslide located on the southern slopes of the Kudłoń Mt.; (**C**) transverse crack—landslide located on the western slopes of the Kiczora Mt.; (**D**) peat bog in the in the upper part of landslide body (the eastern slopes of the Gorc Troszacki Mt.); (**E**) rock formation in the main scarp (the western slopes of the Kiczora Mt.); (**F**) disturbed landslide relief (the southern slopes of the Kudłoń Mt.); (**G**) colluvium of the landslide located on the southern slopes of the Turbacz Mt.

The analyzed area, due to the natural character of the national park, is covered with dense vegetation, which makes it difficult to conduct field studies of landslides (Fig. 2). That is why the opportunities provided by remote sensing methods of geomorphological analysis are so promising.

## Materials and methods

**Figure 3:**
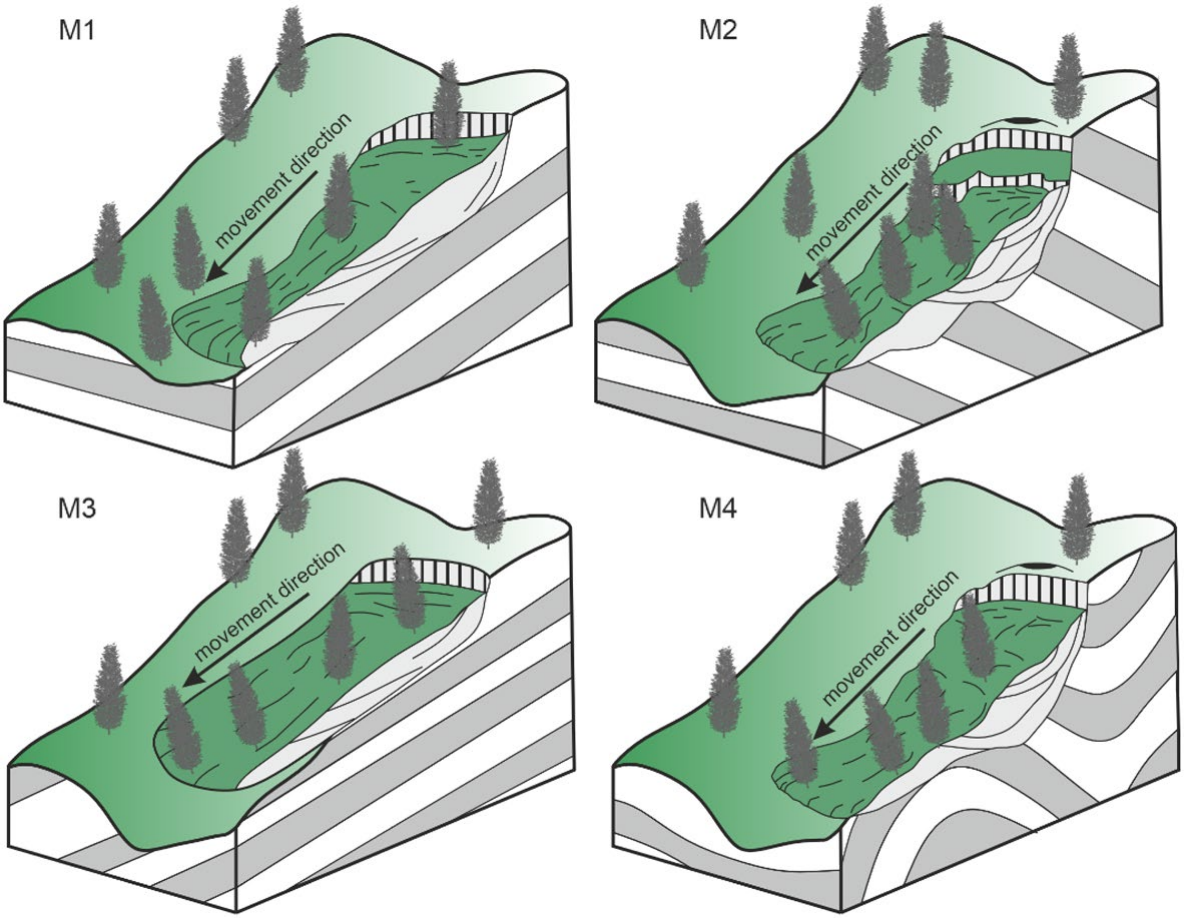
Landslide types according to the relation between the dip of bedding and the direction of the movement: (M1) movement in the same direction, (M2) movement in the opposite direction, (M3) movement perpendicular to dipping, and (M4) complex movement.

The ALS DEM with a spatial resolution of 1 m was used as the main source of data for the presented landslide analysis. The model was published by the Polish Geodesy and Cartography Office. All landslides were visually identified from this high-resolution DEM, manually digitized with the appropriate labels in ArcGIS and verified in the field during extensive geological mapping campaign by Szczęch and Cieszkowski^35^. The landslides were saved in GeoJSON vector format as polygons with two basic attributes shown in Fig. 1, that is, the lithology (L) and landslide type (M). To confirm the correct determination of the location and extent of landslides, field inspections were carried out. The landslides were categorized in two ways:

1. According to the dominating lithology in the landslide detachment zone. We applied the following categories: (L1) thin- and medium-bedded sandstones and shales, (L2) thick-bedded sandstones with intercalations of the thin- and medium-bedded sandstones and shales, and (L3) thick-bedded sandstones. Table 1 shows the relation between the applied categorization and the formal lithostratigraphic units of the Magura Nappe.
2. According to the relationship between the direction of colluvium movement (Fig. 3), interpreted from the DEM and the dipping of the geological beds (based on the fieldworks and geological maps). We applied the following categories after Margielewski^11^ and Migoń et al.^15^: (M1) movement in the same direction as dipping (consequent landslide), (M2) movement in the opposite direction to dipping (insequent landslide), (M3) movement perpendicular to dipping (subsequent landslide), and (M4) complex movement or undetermined situation.

**Figure 4:**
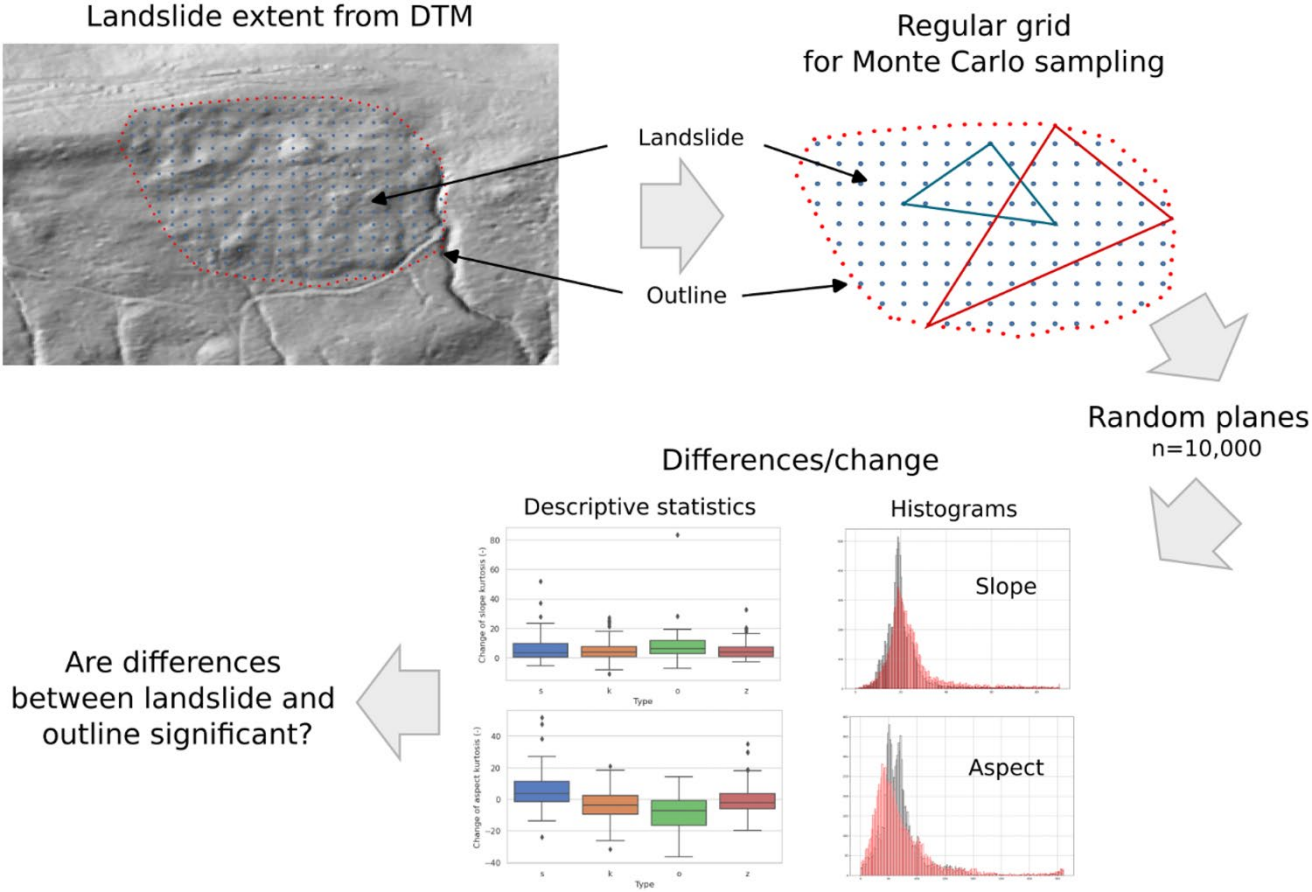
Workflow of the proposed method.

The proposed method assumes that the outline of the landslide represents the original geometric characteristics of the area, while its interior has been deformed as a result of mass movements (Fig. 4). The statistical analysis is based only on the geometric characteristics of the terrain determined from the DEM. Using descriptive statistics, the differences in geometry between the outline of the landslide and its interior are compared in terms of the slope and aspect. The interior part of the landslide is polygonal in nature, while the outline is linear. Because the source material is a raster map, for computational purposes the outline is treated as a line with a minimum width, consistent with the spatial resolution of the DEM, which was 1 m.

The Monte Carlo method^38,39^ was applied with 10,000 sampled random planes (each defined by 3 points) to the landslide body and landslide outline separately (Fig. 4). The slopes and aspects of these planes were determined by calculating the coefficients of equation for each given plane. The goal of the Monte Carlo simulation was to create a large, random data set such that the uncertainties of input data could be mitigated. Then, the coordinates of the maximum slope vector were calculated and converted from the Cartesian to polar coordinate system to obtain the slope and aspect of the plane. The slope and aspect values for the random planes were the basis for creating the planes' distribution, which can be seen in Fig. 4 in the form of two histograms for the slope and aspect. The black histogram represents the planes generated for the outline (contour), while the red histogram indicate the inside of the landslide. For each of the distributions (black and red), descriptive statistics were determined, and then the difference between them was calculated. The obtained difference was the basis for conducting statistical analyses.

We limited the analysis to first order derivatives^40^, but we calculated them for the first four central moments, that is, the mean, standard deviation, skewness and kurtosis^26^. The kurtosis values were calculated according to Fisher's definition with normal equal to 0 for normal distribution. In the case of the aspects, it was necessary to shift the value to the center (180 degrees), to minimize the edge effect at 0 and 360 degrees. The data for the outline and interior of the landslide were shifted to around 180 degrees using the modal value and modulo operation (360 degrees in this case). The average value was not suitable for this purpose due to the cyclic nature. The ANOVA test with the post-hoc Turkey Honest Significant Difference (HSD) test were used to determine if the differences between the landslide and its outline are statistically significant. Using ANOVA, we tested the general differences between the data sets. The Turkey's HSD test was performed only when the ANOVA showed a statistically significant difference between the statistics. For each of the parameters (slope and aspect), the statistical differences were analyzed in this case between all pairs of three lithology types (L) and four landslide types (M). It was therefore possible to make an analysis considering the impact of the geomorphological factors on the occurrence of landslides. All calculations were carried out in the Python environment using scripts and Jupyter notebooks created by the authors with SciPy, geospatial and visualization libraries.

## Results

### Landslides in the Gorce National Park

In the Gorce National Park, we encounter rotational, translational and complex landslides^41^. Within the study area, 364 landslides were identified. They range in size from 0.3 to 430.0 ha. The largest, repeatedly renewed landslide is located on the southern slopes of Kudłoń Mt. Most often, the size of the identified landslides does not exceed 5 ha, and only 20 of them cover an area larger than 20 ha. The landslides are unevenly distributed and cover approximately 10% of the study area, in total about 22 km^2^ (Fig. 1). Some of the identified and verified landslides are still active. Out of all the landslides, 94 have a lithology of L1 type, 139 of L2 and 131 of L3 (Table 1). As for the slides type, 139 of the M1 type landslides are identified, followed by 48 of M2 type, 113 of M3 and 64 of M4. The dominant types of lithology are L2 and landslide movement type M1. The spatial distribution of the individual types of lithology and types of landslides is shown in Fig. 1.

### Landslide geometry changes

**Figure 5:**
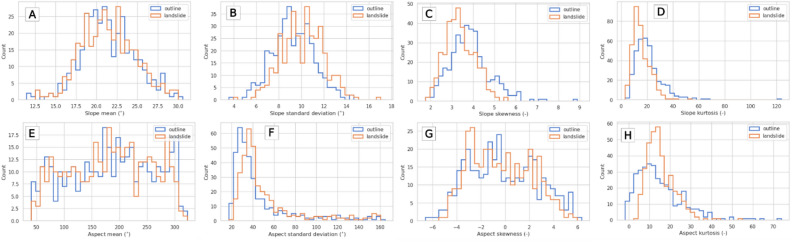
Slope (A–D) and aspect (E–H) histogram comparison for pre-landslide (outline) and landslide descriptive statistics.

The geometry of the landslide outline was treated as a reference (no-landslide) in relation to the area of the landslide itself. A detailed statistical analysis of two landslide geometrical parameters (slope and aspect) was carried out to identify differences between the outline of the landslide and its interior. Changes in the mean values show no visually significant differences (Fig. 5A,E). The situation is different in the case of standard deviations, where an increase in value can be seen after a landslide (Fig. 5B,F). The skewness and kurtosis for the slope decreases (Fig. 5C,D), while the histogram of the aspect kurtosis changes its distribution (Fig. 5H).

The results presented in Fig. 5 do not differentiate landslides due to the lithology and type of landslide, and are presented for visual interpretation. The results of the statistical analysis of the changes in the geometric characteristics of the landslides are presented for lithology type in Table 2, and for landslide movement type in Table 3.

**Table 2:** Differences of descriptive statistics between the landslide (N = 364) and its outline for lithology types. Statistically significant differences at the 0.05 alpha level of importance are underlined.

	Statistic	ANOVA		Turkey's test		
		F	p-value	Lithology pair	HSD	p-value
Slope difference	Mean	0.752	0.472	–	–	–
	Standard deviation	1.700	0.184	–	–	–
	Skewness	5.001	0.007	L1–L2 L1–L3 L2–L3	0.295 0.127 0.168	0.006 0.386 0.121
	Kurtosis	5.285	0.006	L1–L2 L1–L3 L2–L3	3.442 0.753 2.689	0.011 0.803 0.029
Aspect difference	Mean	2.038	0.132	–	–	–
	Standard deviation	6.070	0.003	L1–L2 L1–L3 L2–L3	3.993 2.052 1.941	0.002 0.185 0.144
	Skewness	0.197	0.821	–	–	–
	Kurtosis	3.606	0.028	L1–L2 L1–L3 L2–L3	2.105 4.376 2.271	0.403 0.023 0.264

**Table 3:** Differences of descriptive statistics between the landslide (N = 364) and its outline for landslide movement types. Statistically significant differences at the 0.05 alpha level of importance are underlined.

	Statistic	ANOVA		Turkey's test		
		F	p-value	Movement type	HSD	p-value
Slope difference	Mean	0.375	0.771	–	–	–
	Standard deviation	0.243	0.867	–	–	–
	Skewness	2.719	0.045	M1–M2 M1–M3 M1–M4 M2–M3 M2–M4 M3–M4	0.343 0.131 0.032 0.212 0.311 0.099	0.039 0.491 0.988 0.353 0.094 0.757
	Kurtosis	3.047	0.029	M1–M2 M1–M3 M1–M4 M2–M3 M2–M4 M3–M4	4.630 0.600 0.410 4.031 4.221 0.190	0.019 0.953 0.987 0.056 0.053 0.999
Aspect difference	Mean	2.080	0.103	–	–	–
	Standard deviation	5.965	0.001	M1–M2 M1–M3 M1–M4 M2–M3 M2–M4 M3–M4	5.418 2.161 0.552 3.256 5.969 2.713	0.003 0.208 0.967 0.150 0.001 0.108
	Skewness	0.576	0.631	–	–	–
	Kurtosis	25.983	0.000	M1–M2 M1–M3 M1–M4 M2–M3 M2–M4 M3–M4	5.117 9.982 2.422 15.099 7.540 7.559	0.051 0.000 0.395 0.000 0.002 0.000

Statistical analysis of the differences between the inside and outlines of the landslides (Table 2) showed the L1 group as significantly different (alpha level of importance: 0.05) to the L2 group in terms of the slope skewness and kurtosis. Therefore, landslides developed on deposits containing shale packets and/or medium-bedded sandstones are characterized by a different morphology than those developed on deposits containing thick-bedded sandstone with medium-bedded sandstones or shales. Interestingly, in terms of the slope there is no significant difference between the L1 and L3 groups. Regarding the aspect, there is a difference in terms of the standard deviation between thin- and medium-bedded sandstones with shale-related landslides (L1) and thick- and medium-bedded sandstones with shale-related landslides (L2). Moreover, there is a significant difference between the L1 group and the thick-bedded sandstones-only-related group (L3); however, this is visible only in kurtosis. In general, for the lithology types, statistically significant differences do not appear when analyzing changes in the mean slope and aspect, but they appear in kurtosis. The results for kurtosis perfectly complement each other, which means that when analyzing the slope and aspect, we have statistically significant differences for all lithology combinations. Consequently, we can statistically distinguish the lithologies of slope kurtosis for L1–L2 and L2–L3, and the aspect kurtosis for L1–L3 (Table 2).

The differences were also analyzed in landslide movement related to the bedding dip categories. The M1 (movement with dip) and M2 (movement opposite to dip) show significant differences were only obtained for this pair for all statistics. Only for the kurtosis of aspect differences is the p-value on the verge of the significance level. All pairs, apart from M1–M4, show statistically significant differences for the kurtosis of the aspect change. The M1 and M3 types (movement parallel to dip) as well as M2 and M3 differ in terms of the kurtosis of aspect.

As in the case of lithology, statistically significant differences do not appear when analyzing changes in the mean slope and aspect, but they appear in kurtosis. The most interesting observation is that regardless of the analyzed factor (lithology or type of movement), the general significance of differences for individual descriptive statistics has the same pattern. In both cases, the differences for the slope are significant only at the skewness and kurtosis. Moreover, in both cases, the differences for the aspect are significant in the standard deviation and kurtosis, but insignificant in skewness. This means that, on a general level, the slope and aspect contain similar information about a significant change in geometry. They differ on the level of detail, of course, but that is obvious because of the different categories.

## Discussion

### Landslide database inventory

The presented study concerns the identification of landslides throughout the Gorce National Park, including its buffer zone. The first result of the work was the creation of a vector database with the geometry of the landslides and their attributes related to the lithology and geomorphology. The choice of a national park as the study area ensures that the anthropogenic impact is minimal^42^. Anthropogenic pressure is an important problem in landslide areas. Gorczyca et al.^43^ identified 572 landslides in the Łososina Dolna Commune (Polish Carpathians) with a mean area of 2.3 ha and maximum size of 45 ha. The landslide density index for that area, which is the relationship between the number of landslides and the area, was 8 landslides per km^2^. Sikora^14^ identified 183 landslides in an area of 61 km^2^ in the Western Outer Carpathians, corresponding to a landslide density index of 3. We identified 364 landslides in an area of 236 km^2^, which corresponds to a landslide density index of 1.5. This relatively low rate can be explained by the natural character of the area and the fact that the entire area was analyzed, rather than just the landslide areas. To the best of our knowledge, this is the first such complete study for the area. We have also confirmed that digital terrain models can be a reliable source of information for landslide identification.

While determining the extent of landslides based on LiDAR data was not difficult, assigning lithological types and landslide movement types to individual features was a challenge. To address this issue, we used the detailed geological mapping^35^, to assign a dominating lithology type to the landslide. Assigning a movement type was done based on morphology observations on DEM associated with the bedding strike and dip data measured in the landslide vicinity. Landslides that remain in contact with stream channels are constantly active, as they are under the influence of continuous fluvial erosion^44^. The intensity of colluvium erosion near the rivers in the Outer Carpathians was found to be strongly dependent on the water levels in the adjacent stream^45^. We also observed a similar phenomenon, with a significant number of landslides in the immediate vicinity of watercourses in the Gorce National Park study area.

### Quantitative description of landslide geometry

The use of a DEM to identify landslides is not an innovative approach. The landslide area is recognized by changing the texture of the terrain caused by mass movements leading the surface to undulate. This is a highly effective method, but not without subjectivity. In the proposed method, we attempted to quantify the geometric features of the landslide using the differences of descriptive statistics of the slope and aspect calculated inside and on the outline of the landslide. The data were sampled similarly to Dou et al.^46^ and Li et al.^47^ in a regular grid, as landslide modeling in the Carpathians is sensitive to random sampling^48^.

Süzen and Doyuran^49^ proposed the concept of seed cells that represent the undisturbed morphological conditions before landslide occurs. Seed cells are defined by adding a buffer zone to the crown and flank areas of the landslide. Our approach of creating a thin outline (with a width of 1 m equal to the resolution of the raster) around the landslide representing undisturbed terrain is much simpler to implement and scale. One of the problems of the proposed method is the occurrence of artifacts when the three random points forming the plane are collinear^50^. The lack of adopted threshold values may result in approaching the extreme slope values of 0 or 90 degrees. These outliers, however, were easy to filter and in subsequent implementations of the model, such artifacts can be eliminated already at the stage of triplet plane generation.

Our research shows that the identification of landslide types is visible only after using descriptive statistics of higher orders. On rare occasions, the third (skewness) and fourth (kurtosis) order moments are used to statistically describe the geometric features of landslides. There are exceptions where higher order moments are used^51^. Li et al.^47^ utilized a similar 4-moment method (from mean to kurtosis) to the one we employed, but to describe landslide zones as opposed to individual landslide as one feature.

**Figure 6:**
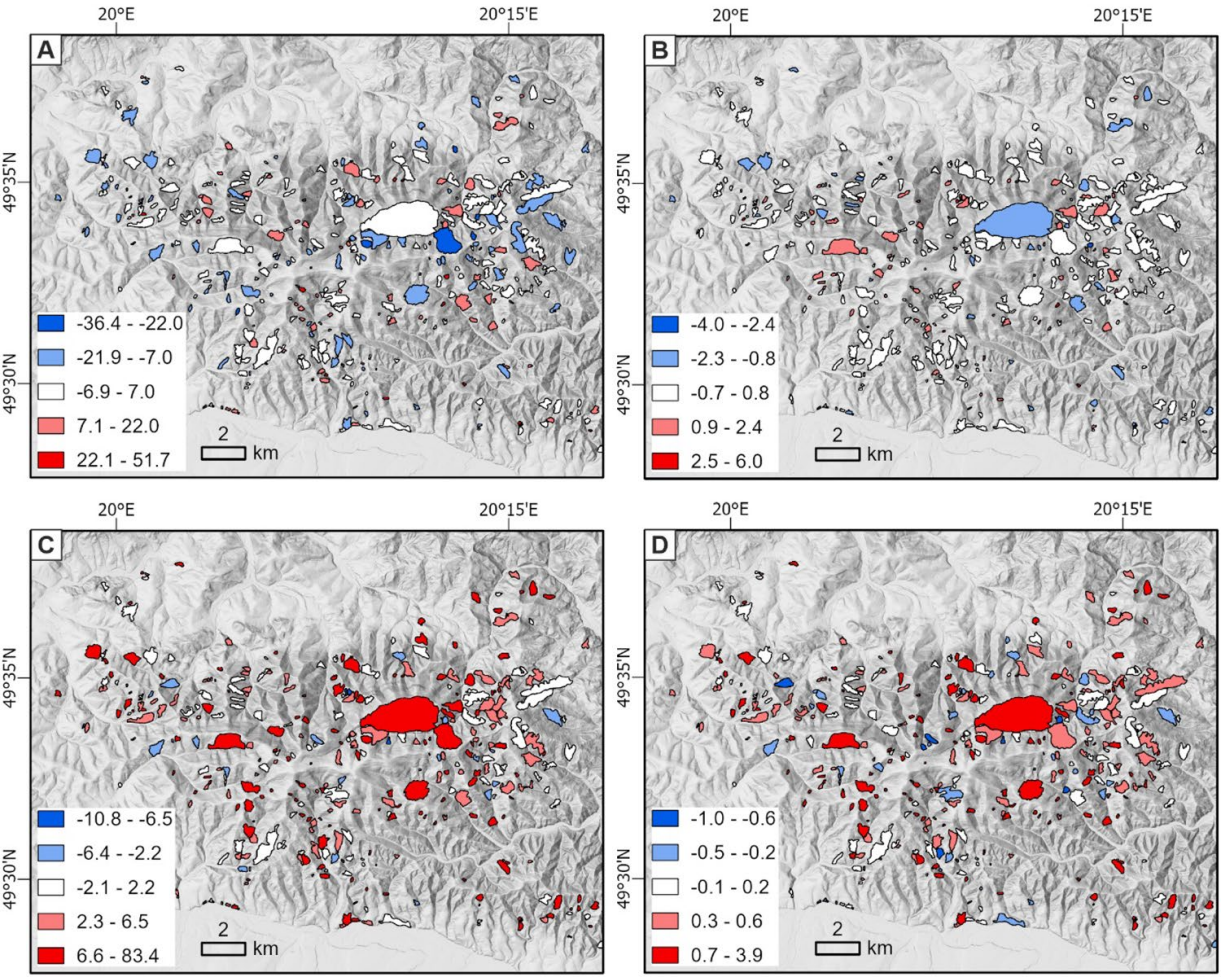
Map of the descriptive statistics' differences between the landslide outline and its interior: (**A**) kurtosis of aspect, (**B**) skewness of aspect, (**C**) kurtosis of slope, and (**D**) skewness of slope.

Skewness is a measure of asymmetry. Kurtosis is a measure of how similar a given distribution is to a normal distribution. The aspect skewness is symmetric (Fig. 5G), while the slope kurtosis (Fig. 5D) and aspect standard deviation (Fig. 5F) are right-skewed. There is also a visible slope skewness decrease as an effect of landslide terrain deformation. This decrease in the slope kurtosis means that the slope values' distribution became closer to a normal distribution. In the case of the aspect, this did not occur, but in both cases (Fig. 5D,H) the kurtosis was more concentrated around the value of 15. Goedecke^52^ similarly found that the skewness and kurtosis of landslide slopes and adjacent areas differ significantly. The spatial distribution of differences in the third and fourth central moment (skewness and kurtosis) are presented in Fig. 6. While the aspect shows no clear patterns (Fig. 6A,B), there is a strong similarity between the results for the skewness and kurtosis with the slope (Fig. 6C,D). The scales of all graphs have been calibrated to the center point 0 represented in white. The clear similarity of these graphs may indicate a deeper relationship between the skewness and kurtosis, although a detailed analysis is beyond the scope of this study.

It can be observed that while negative values predominate for the aspect (Fig. 6A,B), there are significantly more positive results for the slope (Fig. 6C,D). Skewness and kurtosis present more valuable information about landslide geometry than the mean and standard deviation. When making statistical analyses, it is worth reaching for these higher central moments.

### Lithology and structural geology

**Figure 7:**
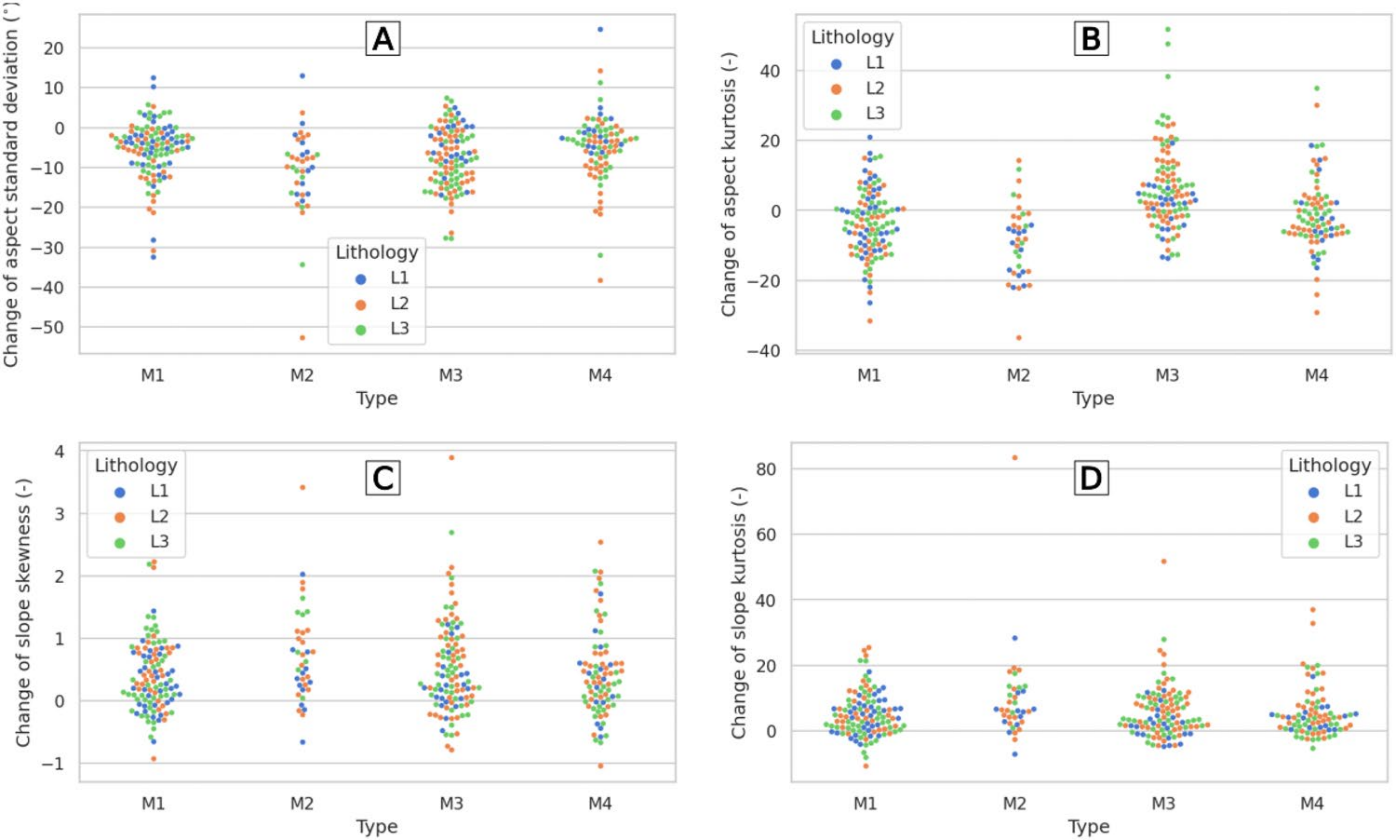
Scatterplot with categorical variables (swarm plot) showing the differences in lithology (L1–L3) and landslide movements (M1–M4): (**A**) standard deviation of aspect, (**B**) kurtosis of aspect, (**C**) skewness of slope, and (**D**) kurtosis of slope.

Detailed results, considering the types of lithology and types of landslide movements, are presented in Fig. 7. The Monte Carlo method^53^ enabled improved statistical description of the landslide and its outline by sampling points from the terrain. According to Keefer^23^, the correlation between landslides and the slope steepness was strongly positive, but for the lithology the correlation was not clear. Huang et al.^13^ showed that the lithology is a more crucial factor influencing landslide susceptibility than the slope or aspect. Our results do not provide grounds for such unambiguous conclusions, but the lithological differences are visible in the statistical results. There is a difference in the statistics calculated for different types of lithology. In particular, the L1 and L2 types differ both in terms of the slope and aspect (Table 2). The landslides developed on shale-dominated formations (L1) are clearly different than these with thick-bedded sandstones intercalated with shales (L2).

A statistically significant difference for the aspect was observed in the kurtosis between the shales (group L1) and thick-bedded sandstones (group L3) (Table 2). The probable reason for this difference can be outliers from type L3 (green dots) visible for the landslide movement types M3 and M4 (Fig. 7B). In the case of the slope, outliers likely also play a key role in differentiating between the L1 and L2 lithologies. For both statistics (skewness and kurtosis), L2-type outliers (orange dots) are highly visible for all landslide motion types except M1 (Fig. 7C,D). Thus, we did not find very clear associations between the identified diverse lithology and landslides. At least, they are not statistically significant when the most common measures such as the mean and standard deviation are used.

Research conducted by Cebulski et al.^54^ on the same DEM data as employed in our investigation but using electrical resistivity tomography on one landslide in the Polish Outer Carpathians showed that lithology had an influence on landslide occurrence. Wódka^42^ presented a suite of characteristic features of landslides in a particular Outer Carpathians area, based on the same data type utilized in this paper (ALS DEM), whereby he found the following types of structures: ripple structures, which are internal scarps and accumulation thresholds, endorheic basins and cracks. These structures were identified in landslides under conditions comparable to groups L2 (thick-bedded sandstones) and M1 (dipping with slope).

The differences are statistically significant between groups of landslides developed on slopes parallel or opposite to the bedding (types M1 or M2), and those developed parallel or sub-parallel to the bedding strike (type M3). There are also differences between types M1 and M2, although these are less clear. There is a statistically significant difference between the skewness and kurtosis of landslides developed on slopes inclined with geological beddings' dip and opposite to it. Considering the aspect, there are statistically significant differences in the standard deviation; however, the most influenced statistic is kurtosis (Table 3). Practically, only the difference between the M1 and M4 types is insignificant. These differences are clearly visible in Fig. 7B, where each type has a different distribution. In the case of the slope, the differences are only between types M1 and M2 (Table 3). However, this difference is not so obvious and can be interpreted as a small variability of the slope skewness differences for M1 versus much more diffuse differences for M2. In kurtosis, this difference is not so visible, but one particular outlying point for M2 may play a key role (Fig. 7D).

The possible slip surfaces of the landslides can be bedding or tectonic structures. Especially in case of landslides with a high content of shales, one can expect slipping according to the bedding. On the other hand, sandstones are prone to gravitational movements due to the presence of tectonic surfaces of anisotropy: joints and faults. This leads to different types of movements like lateral spreading, toppling and sliding^11^. Our results show that substantial amounts of sandstone packets intercalated with shales (lithology type L2) lead to the forming of different morphology in relation to a shale-dominated profile (lithology type L1).

In the Gorce National Park area, we did not find differences in the mean and standard deviation changes for the slope, which would be statistically significant. In our analysis we did not use the topography of the landslide before its occurrence. Instead, we assumed that the outline of the landslide represents the original state of the terrain, which may not always be the case. Lithology and structural geology have a direct impact on the occurrence and geometric features of landslides. We proved statistically significant differences when comparing landslides occurring for different geological categories.

### Applicability of the proposed method

Our first goal was to test a possibly non-subjective and scalable method of statistical description of landslides. The proposed method of using the outline of landslides as a reference area before the mass movement gave positive results, but certainly requires further work. Its advantage is the simplicity of implementation, while the disadvantage is a more difficult interpretation than in the case of using only the scarp crown.

The use of classical descriptive statistics to describe changes in landslide geometry seems substantively justified and, at the same time, straightforward in terms of computation. The proposed method can be easily scaled and automated, which is particularly important in the case of the rapidly growing number of high-resolution digital terrain models. With the increasing use of machine learning in landslide analysis, quantitative information about landslide geometry is important as it makes it easier to define trigger mechanisms^55,56^. Statistically significant differences in the structural context of landslides found for the study area create opportunities for the practical use of this information in other analyses. An important observation was the discovery that more statistically significant differences are seen using skewness and kurtosis than the mean and standard deviation. Therefore, it is worth using the central moments of higher orders, which contain a considerable quantity of valuable information regarding landslides.

## Conclusions

Terrain models with a resolution of 1 m are becoming available for landslide vulnerability analysis and landslide identification^57^. The changes in geometrical statistics before and after a landslide are usually quite spectacular^19^,however, reliable pre-landslide data are not always available. In such cases, the method we propose is one of the potential solutions. Using only the topography deformed by the landslide, we describe the changes in its geometry by taking the landslide outline as the reference geometry. The landslide inventory created for the Gorce National Park in Poland within the present study is the first complete database for this area. The work identified the spatial extents and described the lithological and structural features of 364 landslides. Most of the identified landslides represent the movement direction parallel to the bedding dip azimuth (M1 type), while opposite to the dip azimuth movement (M2) is the least frequent type. This is an expected observation and has a physical justification. A landslide's relationship to the local structural geology (bedding) plays a key role in the modifying morphology in a manner that can be captured by the proposed methodology. The relationship between the lithology and morphology is of secondary importance and is limited to the presence of medium-to-thick beds of sandstones in flysch packets. As a result, the study succeeded in proving the scientific hypothesis that, using only statistical analysis of high-resolution terrain models, it is possible to identify and classify the type and lithology of landslides.

For the statistical description of the landslide geometry, we proposed a new approach using the Monte Carlo method. Descriptive statistics of the slope and aspect were used to estimate changes in the landslide geometry. Significant changes in the landslide geometry are more visible for higher central moments (skewness and kurtosis) than for the first (mean) and second central moments (standard deviation). The use of higher order moments such as skewness and kurtosis for the slope and aspect would improve the classification results, as shown through our analysis. The results obtained confirm the observations of other authors that landslide incidence, morphometry and typology are dependent on the interplay of land characteristics such as lithology. The presented method of statistical description of landslide geometry is a major step toward the automatization of landslide identification and classification using machine learning^58,59^.

## Data Availability

The digital elevation model used in this study is provided by Polish Head Office of Geodesy and Cartography and can be accessed via https://www.geoportal.gov.pl/en/.
